# Editorial Announcement

**Published:** 1917-06

**Authors:** 


### Editorial Announcement.

The increase in cost of paper, printing and postage ought to induce some of our subscribers to settle. Every cent lost and every unnecessary minute on account of back subscriptions being just so much less for the Journal.

